# Adapting and implementing Caring Contacts in a Department of Veterans Affairs emergency department: a pilot study protocol

**DOI:** 10.1186/s40814-019-0503-9

**Published:** 2019-10-10

**Authors:** Sara J. Landes, JoAnn E. Kirchner, John P. Areno, Mark A. Reger, Traci H. Abraham, Jeffery A. Pitcock, Mary J. Bollinger, Katherine Anne Comtois

**Affiliations:** 1 QUERI for Team-Based Behavioral Healthcare, Central Arkansas Veterans Healthcare System, North Little Rock, AR USA; 2 South Central Mental Illness Research Education and Clinical Center (MIRECC), Central Arkansas VA Health Care System, North Little Rock, AR USA; 3 Department of Psychiatry, University of Arkansas for Medical Sciences, Little Rock, AR USA; 4 South Central VA Health Care Network, Ridgeland, MS USA; 5 VA Puget Sound Health Care System, Tacoma, WA USA; 6 Center for Mental Healthcare & Outcomes Research (CeMHOR), Central Arkansas Veterans Healthcare System, North Little Rock, AR USA; 7 University of Washington School of Medicine, Seattle, WA USA

**Keywords:** Suicide prevention, Implementation, Emergency department, Caring Contacts, Veterans

## Abstract

**Background:**

Suicide among veterans is a problem nationally, and suicide prevention remains a high priority for the Department of Veterans Affairs (VA). Focusing suicide prevention initiatives in the emergency department setting provides reach to veterans who may not be seen in mental health and targets a critical risk period, transitions in care following discharge. Caring Contacts is a simple and efficacious suicide prevention approach that could be used to target this risk period. The purpose of this study is to (1) adapt Caring Contacts for use in a VA emergency department, (2) conduct a pilot program at a single VA emergency department, and (3) create an implementation toolkit to facilitate spread of Caring Contacts to other VA facilities.

**Methods:**

This project includes planning activities and a pilot at a VA emergency department. Planning activities will include determining available data sources, determining logistics for identifying and sending Caring Contacts, and creating an implementation toolkit. We will conduct qualitative interviews with emergency department staff and other key stakeholders to gather data on what is needed to adapt and implement Caring Contacts in a VA emergency department setting and possible barriers to and facilitators of implementation. An advisory board of key stakeholders in the facility will be created. Qualitative findings from interviews will be presented to the advisory board for discussion, and the board will use these data to inform decision making regarding implementation of the pilot. Once the pilot is underway, the advisory board will convene again to discuss ongoing progress and determine if any changes are needed to the implementation of the Caring Contacts intervention.

**Discussion:**

Findings from the current project will inform future scale-up and spread of this innovation to other VA medical center emergency departments across the network and other networks. The current pilot will adapt Caring Contacts, create an implementation toolkit and implementation guide, evaluate the feasibility of gathering outcome measures, and provide information about what is needed to implement this evidence-based suicide prevention intervention in a VA emergency department.

## Background

Suicide among veterans is a problem nationally, and suicide prevention remains a high priority for the Department of Veterans Affairs (VA). While rates of suicide have increased for both veteran and non-veteran populations, the suicide rates for veterans are higher than for the general population [1]. In 2016, after adjusting for age and gender, the suicide rate was 1.5 times greater for veterans than for non-veterans [1]. Rural veterans are at even greater risk for suicide; in one study of veterans using VA healthcare, rural veterans’ risk was 20–22% greater than urban dwelling veterans and the use of a firearm was more common in rural suicides [2].

Suicide prevention efforts are usually prioritized in mental health treatment settings. However, about half of those who died by suicide in the USA had no known mental health condition [3], and similar results have been observed in military populations [4]. Focusing on non-mental health settings such as the emergency department thus broadens the scope of suicide prevention and also improves the reach of prevention efforts to a greater proportion of veterans [5], particularly rural veterans who are at greater risk for suicide and who may be less likely to come into a mental health clinic [6, 7]. In general, VA has recently broadened their focus on screening for suicide to include all settings, including the emergency department. The new mandated screening requirements include (a) a primary screen of all patients with the Patient Health Questionnaire (PHQ-9) item 9, (b) a secondary screen for all positive primary screens, and (c) comprehensive suicide risk assessment for all positive secondary screens.

Supporting this approach, national suicide prevention initiatives have highlighted transitions in care, such as discharge from an emergency department, as critical target periods to improve suicide prevention [8–12]. Research supports the assertion that the transition in care following discharge is a critical time period [13]. The majority of suicides occur within 30 days after discharge from the hospital or emergency department, with most occurring within 1 week. In one study, nearly 25% of patients either attempted or died by suicide within 12 months after screening positive for suicidal ideation in an emergency department [14]. Providers need simple and effective interventions to improve care during the critical transition following emergency department discharge [9, 15–17].

Caring Contacts (CC) is a simple and efficacious suicide prevention approach that involves sending patients who are suicidal brief, non-demanding expressions of care and concern at specified intervals over a year or more [18–22]. The theoretical basis for CC and its possible mechanism of action relate to a lack of social connection, a key risk factor for suicide [23–37]. CC have been sent via different modalities, such as postal mail (e.g., letter, greeting card, postcard in an envelope), email, and most recently, text message. In the most frequently used schedule, CC were sent to patients 1, 2, 3, 4, 6, 8, 10, and 12 months after discharge [38].

CC is one of the only suicide prevention interventions that have reduced rates of death by suicide in randomized controlled trials*.* Studies of CC have demonstrated significant reductions in suicide deaths [18, 19], suicide attempts, and suicidal ideation at 1- and 2-year follow-up [20–22]. CC have been found to be feasible and acceptable [39] with military and veteran populations, and effective with active duty soldiers and marines [40]. In a study on veteran perspectives [41], the majority (83%) thought CC could be helpful to veterans who are suicidal. Veterans indicated a preference for receiving postal mail (e.g., letter, postcard in an envelope, or greeting card) over email or text message [41]. A review of studies on CC determined that “repeated follow-up contacts appear to reduce suicidal behavior (p. 32)” [42]. CC were also found to be cost-effective [43]. Strong full trials of CC in the emergency department setting have not been conducted. One RCT ended early when initial results indicated a significantly positive impact on rate of re-presentation for self-harm. However, errors in analyzing data resulted in different final results [44]. Pilots have demonstrated that CC in the emergency department is feasible and acceptable [45, 46].

### Gap in the literature/purpose of study

Given the higher risk of suicide among veterans, and rural veterans in particular, limitations to focusing suicide prevention efforts upon veterans using mental health services, increased suicide risk following transitions in care after visiting an emergency department, and the efficacy of CC, we have proposed to implement CC in the emergency department. CC have not been implemented broadly in VA, despite recommendations for their use. While CC is a simple intervention, the logistics of identifying, sending, and tracking contacts over the course of a year for numerous people is an unmanageable task for individual providers. To our knowledge, no implementation studies have been conducted on how to implement this effective intervention in a real-world setting.

The purpose of this study is to determine how to implement CC in a VA emergency department in collaboration with emergency department staff, suicide prevention coordinators, and other key stakeholders. This project will (1) adapt CC for use in a VA emergency department, (2) conduct a pilot implementation study at a single VA emergency department, and (3) create an implementation toolkit to facilitate spread of CC implementation to other VA facilities. This project is important because not all veterans who experience suicidal ideation or suicidal behavior have mental health diagnoses or engage in mental health services. Focusing on non-mental health settings, such as the emergency department, therefore broadens the scope of suicide prevention, allowing reach to a greater proportion of veterans served by VA. In addition, this project will create an implementation toolkit that includes an implementation guide, a document that includes a step by step process of how to plan for implementation, which can be used inside or outside VA.

### Collaboration

**Fig. 1:**
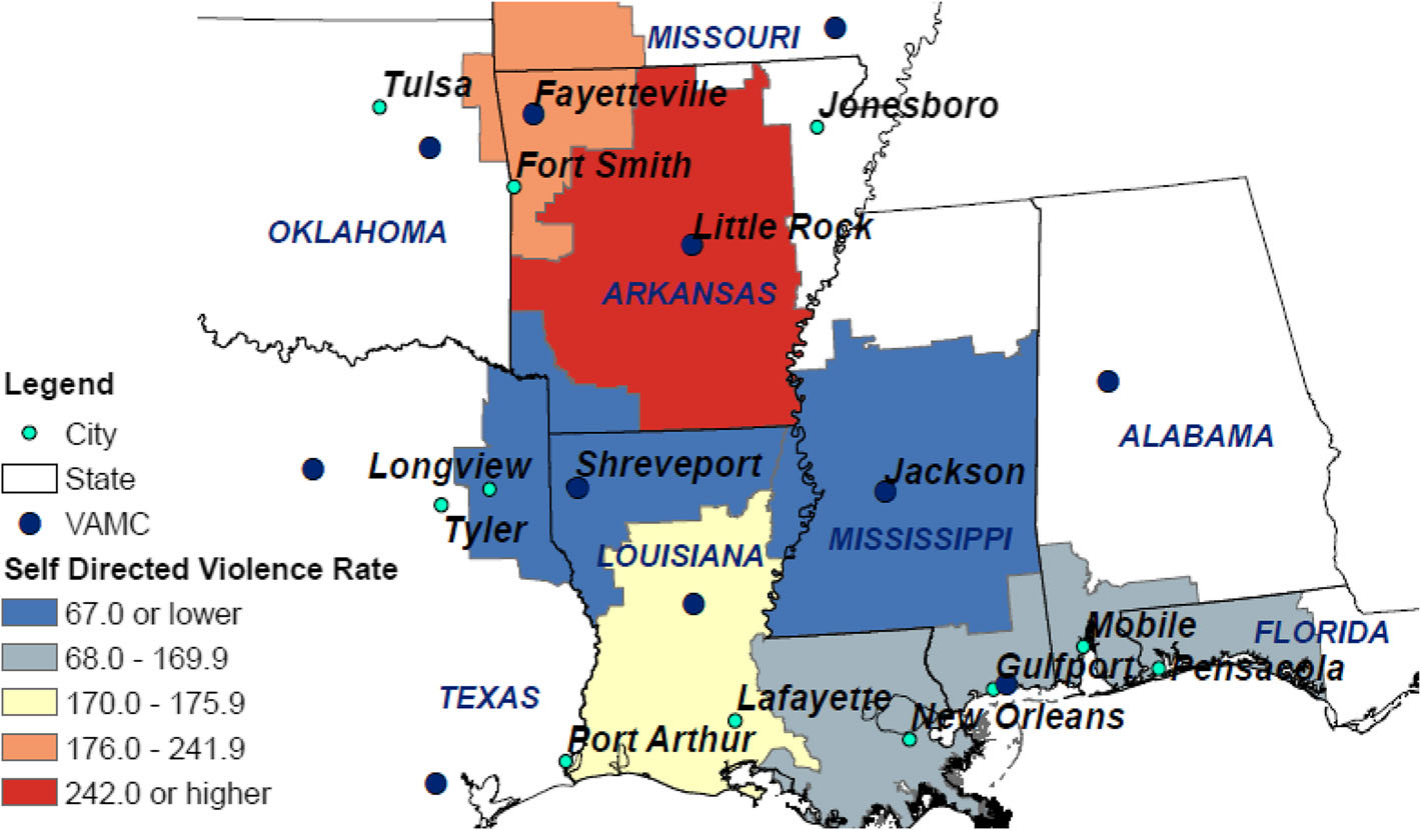
Rates of self-directed violence per 100,000 enrolled veterans by VA submarket in VISN 16, October 2016–Dec 2017

VA is comprised of 18 regions of care; these regions are referred to as Veteran Integrated Service Networks (VISNs). This project is being conducted in collaboration with VISN 16. VISN 16 is the fourth most rural network, with 41% of the 419,374 healthcare users classified as rural or highly rural. It encompasses eight states including Louisiana, most of Arkansas, and parts of Missouri, Oklahoma, Texas, Mississippi, Alabama, and Florida. As for VA generally, suicide and self-directed violence are a challenge for this Network (see Fig. 1). Thus, VISN clinical and operation leadership were ready partners for this unique funding opportunity, described next. Collaboration efforts thus far with VISN leadership have included meeting with the full leadership team to discuss their priorities, collaborating with the Chief Medical Officer (JPA) on the submission and conduct of the grant, and including the VISN mental health lead on the advisory board (see “Methods” regarding the composition of the advisory board).

This project is funded by a VA Quality Enhancement Research Initiative (QUERI) grant. QUERI funds quality improvement and program evaluation studies to support time-sensitive implementation and evaluation efforts needed to improve healthcare for the nation’s veterans [47]. This project received funding through a peer-reviewed QUERI-VISN Partnered Implementation Initiative (PII) grant that requires partnership between VISN leadership (director or chief medical officer) and an implementation expert. The grant provides 1 year of funding for planning and conducting an implementation pilot at a facility within the VISN. The focus of QUERI-VISN PII grants is improving veteran health by rapidly implementing evidence-based practices (EBPs) and planning for scale-up and spread across the VISN. Teams then apply for additional PII funding to facilitate EBP spread across the VISN and other VISNs, with a goal of enterprise-wide scale-up and spread.

The study team also collaborated with the leadership of the pilot facility, located in Central Arkansas, in the planning of the project. Facility leadership was motivated to implement a suicide prevention intervention in the emergency department and provided facility funds to begin planning prior to initiation of the grant. Collaboration efforts with facility leadership have thus far included discussing project goals with the leadership team, presenting at the facility senior leadership retreat to spread knowledge of the project, and identifying a point of contact in the emergency department to lead the local pilot.

## Methods

### Overview

The present project includes planning activities and an implementation pilot at the Central Arkansas Veterans Healthcare System located in VISN 16. Planning activities will include determining what data sources (e.g., self-directed violence, positive suicide screens) are available for the emergency department and how to best access those data, working with the facility mail room to identify appropriate mailing processes, and creating an implementation toolkit. To inform the pilot, we will also conduct qualitative interviews with emergency department staff and other key stakeholders to gather data on what is needed to adapt and implement CC for a VA emergency department setting (e.g., how to identify veterans to receive CC, signatory of CC, schedule for contacts) and possible barriers to and facilitators of implementation. Discussed in greater detail below, planning activities will include assembling an advisory board of key stakeholders in the targeted facility (e.g., emergency department staff, suicide prevention coordinators), veterans, and experts in implementation and CC. Qualitative findings from key stakeholder interviews will be presented to the advisory board for discussion, and the board will use these data to inform decision making regarding implementation of the pilot. Once the pilot is underway, the advisory board will convene again to discuss ongoing progress and determine if any changes are needed to the implementation of the CC intervention.

### Setting

The project setting is an emergency department at a large VA medical center in a Southern, highly rural state. This emergency department employs 15 physicians, two midlevel providers, 56 nurses, and four social workers. An average of 2000 veterans are seen monthly; 200 are for mental health reasons. Psychiatric services are provided by a psychiatric consult-liaison service during the day and by psychiatry residents on call at night.

### Participants

All veterans seen in the emergency department who are clinically appropriate for CC will receive the intervention. As described below, the method for identifying veterans appropriate for CC will be determined during the planning phase. Each emergency department staff’s participation in the implementation of CC will also be determined during the initial planning phase and tailored to their role in the emergency department.

### Implementation framework and strategy

The integrated Promoting Action Research on Implementation in Health Services (i-PARIHS) framework proposes that successful implementation of evidence-based practices is the result of the facilitation of an innovation with recipients in the inner and outer setting context. Successful implementation is defined as the achievement of agreed goals, the uptake and institutionalization of the innovation, engaged stakeholders who own the innovation, and the minimization of variation related to context across implementation settings [48]. Among the i-PARIHS constructs, facilitation is the active ingredient with designated facilitators activating implementation by assessing and responding to the characteristics of the recipients of the innovation within their own settings.

Facilitation is the strategy chosen for implementing CC in the targeted VA facility because of its flexibility and evidence of success [49, 50]. Facilitation is a multi-faceted “process of interactive problem solving and support that occurs in the context of a recognized need for improvement and a supportive interpersonal relationship” [51]. Facilitation has been used nationally across VA to implement a number of different clinical interventions and has been further developed as a strategy through partnerships between VA researchers and VA operational partners [49–52]. Depending upon the complexity of the innovation and need of the sites, facilitation can vary by type and amount offered. For example, it can be provided by a facilitator external to the clinic or medical setting (external facilitation) or through a more complex model of combined internal and external facilitators.

An external facilitator is an expert in implementation who is either a subject matter expert in the relevant clinical areas or has access to one [53]. External facilitators use a variety of strategies to facilitate implementation, including provider education, performance monitoring and feedback, and formative evaluation. Facilitators’ actions are dependent on a facility’s needs and the timing of the implementation process. To adapt to each facility’s clinical context, facilitators select from a range of strategies (described below in Table 1) based upon an assessment of each facility’s needs, and barriers to and facilitators of implementation.

**Table 1:** Facilitation strategies and activities

Strategy	Possible activities
Facilitate local change agent participation	• Help CC staff engage their facility and impacted providers
	• Encourage suicide prevention coordinators to champion CC
Conduct provider education	• Conduct virtual site visits
	• Provide briefings about CC to medical center management and/or VISN mental health leads to ensure they are aware of and supportive of CC
	• Educate CC staff and providers on CC program components
	• Direct CC staff and providers to resource materials
Facilitate stakeholder engagement	• Engage regional and medical center managers directly through presentations about CC
	• Incorporate process of implementation feedback into existing leadership meetings and information dissemination meetings
	• Be available for consultation about the program to regional and local leadership as needed and as identified by local change agents
Facilitate performance monitoring and feedback	• Create reports of CC staff and provider activity
	• Present reports to CC staff and local leadership
Conduct formative evaluation	• Help sites identify possible barriers and facilitators to implementation and address them
Facilitate program marketing	• Support marketing activities

Virtual external facilitation has established effectiveness when used nationally in VA. For example, in one study that implemented a low complexity intervention in which a provider utilized a patient registry to contact patients lost to care, facilitation was conducted via telephone contacts for 6 months and included one to three calls per month, each lasting approximately 30 min. Each facility received an average of 7 h of facilitation [49].

During the pilot, external facilitation will be provided by the first author (SJL). This will allow us to explore the type and amount of facilitation that is needed to implement CC in a VA emergency department. Given the low complexity of CC, we anticipate that virtual external facilitation conducted via telephone will be appropriate.

### Implementation toolkit

During the planning phase and pilot, we will develop a CC implementation toolkit for external facilitators. We anticipate that the CC implementation toolkit will include an implementation planning guide [54, 55], leadership briefings, educational materials (e.g., PowerPoint presentations, FAQs), CC templates, protocols (e.g., for identifying veterans to receive CC, sending CC, documenting CC), guidance for aligning CC with suicide prevention coordinator responsibilities, and instructions on how to access data for monitoring and feedback.

As described below in “Process and outcome metrics”, we will measure the time and activities involved in facilitation during the planning phase to inform development and refinement of the implementation strategy and toolkit. For example, if initial tracking at the start of implementation indicates that completion of the steps identified in the planning guide are not being accomplished, facilitation calls will be scheduled more frequently. Creation of this toolkit will help improve future scale-up and spread, as external facilitators will have the resources needed and can focus on working directly with sites to implement CC.

### Intervention: Caring Contacts

CC will consist of brief messages of care and concern consistent with research methods that obtained beneficial outcomes [20, 21]. Messages will be sent on behalf of a VA provider and documented in the electronic health record (EHR). Consistent with documented veteran preference, CC will be printed on a postcard and sent in a sealed envelope via postal mail. The envelopes will be colored like a greeting card to increase the likelihood they will be noticed and opened by recipients. Although we considered sending CC via text message, we determined that we would be unable to solve technology issues during the 1-year project timeframe. We will continue to explore this option during the planning phase and include later if feasible.

During the planning phase and pilot, we will determine the method of identifying veterans to receive CC, any changes to wording of the messages in the CC, the signatories of the message (i.e., which VA provider will be authoring the message), and the schedule. The schedules in the literature vary depending on the setting. For example, in studies where CC are sent following discharge from inpatient or outpatient mental health, CC were sent within the first month and then at 2, 3, 4, 6, 8, 10, and 12 months [20, 22]. In pilot studies where CC were sent following discharge from the emergency department, CC were sent within days of discharge and more frequently than monthly [45]. We will focus on methods that will allow scale-up of CC in a way that remains meaningful to the patient while minimizing provider burden.

Protocols have been developed to organize responses to patient replies to CC (e.g., expression of thanks, indications of risk, requests to cease sending CC). We will set up a dedicated phone line to be used for CC; in line with VA policy for voicemail messages, this phone line will have a message indicating when someone is available to return their call and to call 911 or the Veterans Crisis Line if they are at immediate risk. We will also set up a dedicated mail slot to receive mail responses. Each day, emergency department staff will check the voicemail and mail slot and respond to messages in line with facility guidelines (e.g., following crisis response plans as needed, returning calls of thanks). Requests to stop sending CC will be documented in the EHR and all future CC will not be sent.

### Advisory board

We will assemble an advisory board of key stakeholders and experts. The advisory board will include the primary point of contact for the emergency department (the nurse manager), additional emergency department staff (e.g., physician, social worker, and health technician), suicide prevention coordinator, veterans, VISN mental health lead, experts in implementation (JEK, SJL), and an expert in CC (KAC). If other relevant stakeholders are identified in the qualitative interviews, they will be invited to the advisory board.

The advisory board will follow an implementation planning guide to complete this process [55]. The implementation planning guide walks key stakeholders through the process of decision making and planning needed to implement a practice. This includes decisions and tasks such as identifying participating staff, determining the start date, and creating a plan to engage and train staff. The advisory board will concurrently adapt the planning guide to be specific to CC to be used at subsequent sites following the pilot. This will include adding relevant questions identified during the planning process (e.g., determining who will receive CC, specifying the logistics of sending CC). The advisory board will review summaries of qualitative data from stakeholders (described below) and use that information to make decisions about CC as described above (e.g., message, signatory, schedule). In addition, the advisory board will monitor process and outcome metrics (described below) and inform the identification of strategies the facilitator can apply if CC are not implemented with fidelity to the model. This will be an iterative process in which initial decisions are made based on the best data available, applied, and modified if anticipated outcomes are not achieved [56].

### Process and outcome metrics

In addition, the sample size will vary based on decisions made by the advisory board. For example, if the advisory board recommends sending CC to all veterans who screen positive on the primary screen (PHQ-9, item 9), the sample will be larger than if they recommend sending CC to those who are positive on the secondary screen.

We will use RE-AIM [57] as an analytic framework. RE-AIM is a common framework used to evaluate implementation outcomes. RE-AIM examines five dimensions related to implementation and impact of an intervention: reach into the target population, effectiveness of the intervention, adoption by the setting, implementation consistency or fidelity, and maintenance over time. In Table 2, we list the process and outcome metrics we propose to use to evaluate the implementation of CC. We will use the planning phase to further refine these metrics; we will determine the frequency with which these data are available and updated and how network leadership prefer to access that data for VISN 16. We will work with our VISN lead and advisory board to determine if other measures are needed.

**Table 2:** Process and outcome metrics

Process/outcome metric	Definition
Reach	Number and % of veterans receiving CC per facility
Adoption	Number and % of ED providers who identify patients as appropriate for CC per facility
Implementation fidelity	Content of CC
	Date sent and alignment with schedule
	Responses to veteran replies consistent with protocol
Maintenance	To be determined in the planning phase
Effectiveness: suicide-related behavior	Self-directed violence rate
	Injury rate
	Fatality rate
Effectiveness: service utilization	Outpatient mental health encounters
	Outpatient health/other encounters
	Emergency services for mental health
	Inpatient services for mental health
	Emergency services for health/other
Effectiveness: mental health SAIL metrics	PMED1 (% of patients with a mental health diagnosis who have a mental health evaluation and management encounter)
	HRF2 (% of patients with a new or reactivated high-risk flag (HRF) who received at least four mental health visits within 30 days of flag initiation)
Cost	Cost of implementing CC
	Cost of providing CC
	Downstream healthcare utilization costs
Staff perspective	Key informant interviews focused on staff perspective of CC
Veteran perspective	Key informant interviews focused on veteran perspective of CC

#### Reach

Reach will be evaluated by the number and percentage of veterans receiving CC in the emergency department. This data will be collected using the spreadsheet created for tracking CC (for each veteran, whether the CC was sent and date sent will be recorded for each time point). The data in the spreadsheet will be compared to the number of eligible veterans as identified by EHR data. The criteria for determining whether a veteran is appropriate for CC (e.g., reporting suicidality, presenting with any mental health concern) will be determined during the implementation planning process. We expect the numbers and percentage of veterans receiving CC will increase over time and will collect these data for a 90-day period post implementation.

#### Adoption

Adoption will be evaluated by the number and percentage of emergency department providers who adopt CC by identifying patients as appropriate for CC. This data will be collected through the EHR. We will compare the names of providers who identify veterans to receive CC to a list of all possible providers (e.g., if nurses are identified as the providers to send CC, we will pull names of nurses who send them and compare them to the list of all nurses who work in the emergency department). We expect the numbers and percentage of emergency department providers using CC will increase, and will collect these data for a 90-day period post implementation.

#### Implementation fidelity

We will measure fidelity to the evidence-based practice through review of the EHR. The staff member identified to send CC will document each contact in the EHR; this will include the content of the contact and date of contact. Implementation fidelity will be defined by (1) adherence to the schedule for sending CC and (2) whether the appropriate CC template was used. The schedule for sending CC will be determined during the planning and pilot; one possible schedule is the most frequently used schedule in CC research studies (months 1, 2, 3, 4, 6, 8, 10, and 12). Templates will also be created during the planning phase to be used during the pilot; these templates will include messages that are caring and non-demanding. For each veteran receiving CC, we will track whether each CC was sent according to schedule (Y/N) and whether the appropriate template was used (Y/N). These data will be collected for a 90-day period post implementation.

In addition, each response to veteran replies to CC will be documented in the EHR. We will code (1) whether veterans received a response when they should have (given the response protocol), (2) the timeframe of the response, and (3) what the response included (e.g., expression of caring, referring to available resources, answering a question, providing management of acute risk). Coding categories for the responses are not mutually exclusive.

#### Maintenance

Sustainability measures, typically repeated measures of Reach, Effectiveness, Implementation, and Adoption, will not be collected due to the latency of the pilot study.

#### Effectiveness: suicide-related behavior

To examine the feasibility of collecting data to evaluate the effectiveness of CC, we will obtain data on suicide-related behaviors from EHR data and from VA’s Suicide Prevention Applications Network (SPAN). ICD codes contained in the EHR for suicide-related behaviors are notoriously incomplete. Therefore, the VA’s suicide surveillance data (SPAN) will be used to improve data quality. SPAN receives data from suicide prevention coordinators relating to suicidal ideation and suicidal behavior of veterans. Data are submitted to the VHA Support Service Center (VSSC) and are cleaned, processed, and managed by statistical staff and program analysts at the VISN 2 Center of Excellence for Suicide Prevention. Data can be accessed by each facility and combined into a VISN-level report. During the planning phase, we will determine the frequency with which updated data is available.

We will measure suicide-related behavior, defined in SPAN as (1) non-suicidal self-directed violence (preparatory), (2) suicidal self-directed violence (preparatory), (3) suicide, (4) suicide attempt (with injury, with injury and interrupted by self/other, without injury, without injury and interrupted by self/other), and (5) undetermined self-directed violence (fatal, preparatory, with injury, with injury and interrupted by self/other, without injury, without injury and interrupted by self/other). These data will be obtained from SPAN. Documentation of a suicide-related behavior in either SPAN or the EHR will be counted as an event. Given that this is program evaluation, we will not administer patient-level measures of suicidal ideation or behavior.

#### Effectiveness: service utilization

We are measuring service utilization to examine the impact of CC on veteran outcomes that are proximal to suicidal behavior. Changes in service utilization, such as increased outpatient mental health visits and decreased emergency room visits, will allow us to examine the impact on patient care. We will obtain this data from the EHR.

Changes in service utilization can also be viewed as implementation outcomes for the healthcare system. For example, decreased emergency department visits may reduce cost of patient care to the system. This will be accounted for below in the cost metrics.

#### Effectiveness: mental health SAIL metrics

VISNs and VA facilities are evaluated based on their performance on a variety of measures, including SAIL metrics. SAIL, which stands for Strategic Analytics for Improvement and Learning Value Model, is a system for summarizing hospital system performance within VA. SAIL assesses 25 quality measures in areas such as death rate, complications, and patient satisfaction, as well as overall efficiency and physician capacity at individual VA medical centers [58]. This funding request required inclusion of SAIL metrics of priority to the healthcare system. In partnership with our VISN leadership, we identified two relevant mental health SAIL metrics that may be impacted by CC.

We classified SAIL metrics as effectiveness outcomes as this is the way it is used by the healthcare system. The first mental health SAIL metric is the percentage of patients with a mental health diagnosis who have a mental health evaluation and management encounter (PMED1). The second is the percentage of patients with a new or reactivated high-risk flag (HRF) who received at least four mental health visits within 30 days of flag initiation (HRF2). We expect an increase in both metrics, as CC is expected to increase engagement. We will also examine the impact on HRF1 (percentage of patients with a new or reactivated HRF with a documented safety plan within 7 days of flag initiation) and HRF5 (percentage of patients with a new, reactivated, or continued HRF who receive a case review within 100 days of flag initiation).

#### Cost

We will collect implementation and intervention cost data to support a future budget impact analysis. Implementation activities will be categorized and assigned a time and personnel ID; these activities will be tracked by the external facilitator using the BH QUERI Time Tracker. Intervention costs will include materials and staff time involved in sending the CC; these activities will be tracked by study staff and the ED point of contact. Downstream healthcare utilization costs will be extracted from the EHR as part of a future budget impact analysis.

#### Staff perspective

We will conduct key informant interviews before, during, and after the implementation to gather employee perspectives on the implementation of CC. We will conduct qualitative interviews with emergency department staff, suicide prevention coordinators, and other relevant stakeholders (e.g., outpatient mental health providers) (*N* = 15). These interviews will focus on questions relevant to adapting and implementing CC in a VA emergency department. Questions will include how to identify veterans to receive CC, who should be the signatory of the CC, the schedule for sending, how to address the logistics of sending CC, and possible barriers to and facilitators of implementing CC. We will conduct additional interviews during the pilot about the progress of implementation. These interviews will also include information about veterans’ responses to CC and staff replies.

#### Veteran perspective

We will also conduct key informant interviews during implementation with veterans receiving CC (*N* = 5) to gather patient perspectives on receiving CC. These will focus on whether the CC were received, their perceived helpfulness, and patient perspectives on the modality, content, frequency, and signatory of CC. These interviews will also inquire about whether veterans responded to the CC and their perception of the reply to their response (e.g., if they called in response to the CC, what did they think of the provider reply to that phone call). Interviews with veterans will occur 6 months after they receive their first CC.

### Planned analyses

#### Quantitative analysis

One purpose of this pilot study is to assess the feasibility of the collection of specific data elements for quantitative analysis. Given the limited period of data collection and the specific aims, we will report only summary statistics for all quantifiable measures over the 90-day post-implementation period. 

#### Qualitative analysis

Rapid analytic techniques informed by Hamilton [59] will be used to quickly produce analytic findings for use by the study team and advisory board. All interviews will be digitally recorded and transcribed verbatim. A qualitative analyst will review each transcript and summarize interview content using a template (i.e., an electronic document containing a table with domains and categories based in implementation goals). Content from the individual templates will be aggregated in a summary template, and these findings will be used to adapt implementation of CC to the emergency department. To establish rigor, all individual templates will be audited (i.e., a second analyst will read the transcript and review completed templates to ensure that content was summarized accurately). A template rubric will also be developed to define template domains and categories. The rubric will ensure consistency in how content is categorized in the templates, akin to establishing agreement among coders [60].

### Ethics

This program evaluation project, Implementing Caring Contacts for Suicide Prevention in Non-Mental Health Settings (#1301402-1), was reviewed by the Central Arkansas Veterans Healthcare System (CAVHS) Institutional Review Board (IRB) and received a determination of non-research. This evaluation will involve direct data collection from VA employees at CAVHS and veterans who have visited the CAVHS emergency department, as well as indirect data collection through the EHR. Prospective participants in direct data collection will receive with an information sheet and have the opportunity to discuss the evaluation with study staff. Verbal consent will be obtained, as the interviews will take place via telephone and written forms may be the only paper link between participant name and their information.

All participants in this evaluation will be assigned an ID to be used on any forms and in the data management spreadsheet. De-identified data will be stored securely in a dually locked area. Electronic data will be stored in a password-protected database on a server only accessible by the study team. The complete dataset will reside at the Central Arkansas Veterans Healthcare System, and only evaluation staff members will have access to the data.

## Discussion

CC is an efficacious suicide prevention intervention and its implementation in the emergency department allows for a broader reach to veterans and targets a critical transition period with high risk for suicide. This project will include planning activities and a pilot in a VA emergency department. Planning activities will include determining feasibility of accessing data needed and creating an implementation toolkit. A key component of the toolkit will be the implementation guide, a document that leads a team through the decision-making process needed to implement CC in their setting. The toolkit will be created for use by facilitators for future scale-up and spread.

A strength of this project is the network and facility-level collaboration. This demonstrates a leadership commitment to the project, provision of resources, and support for scale-up and spread should the pilot go well. The project also includes collaboration with key stakeholders across the facility, including participation in qualitative interviews and the advisory board. Working directly with emergency department staff and others across the facility, such as suicide prevention coordinators, will ensure that the implementation guide and toolkit created will address relevant topics. The creation of the implementation planning guide is an important product, as the guide will walk stakeholders in any setting through the process of determining how to implement CC.

The primary limitation to this project is the inclusion of only one emergency department in the pilot. This limitation is mitigated by the ability of the implementation planning guide to be used in other locations. In addition, if this pilot is successful, next steps will include spread across the VISN, which will include seven other emergency departments, before spread to other VISNs. This will allow for an iterative process in developing the implementation planning guide and toolkit. Another limitation is the lack of patient-level outcome assessments outside of what is collected in the EHR given that this is program evaluation and focused more on how to implement the intervention. Regarding outcome metrics, it is unclear whether an intervention in the emergency department will impact the mental health SAIL metrics identified, as these metrics are for the entire facility.

In conclusion, positive findings from the current project will inform future scale-up and spread of this innovation to other VA medical center emergency departments across VISN 16 and other VISNs. The current pilot will adapt CC, create an implementation toolkit and implementation guide, evaluate the feasibility of gathering the selected outcome measures, and provide information about what is needed to implement this evidence-based suicide prevention intervention in a VA emergency department.

## Data Availability

Not applicable.

## Abbreviations

CAVHS: Central Arkansas Veterans Healthcare System
CC: Caring Contacts
CeMHOR: Center for Mental Healthcare and Outcomes Research
EHR: Electronic health record
HRF: High-risk flag
i-PARIHS: Integrated Promoting Action on Research Implementation in Health Services
MIRECC: Mental Illness Recovery Education Clinical Center
PII: Partnered Implementation Initiative
PMED1%: MH-diagnosed veterans who had an evaluation and management visit
QUERI: Quality Enhancement Research Initiative
RE-AIM: Reach, effectiveness, adoption, implementation, and maintenance
SAIL: Strategic Analytics for Improvement and Learning
SPAN: Suicide Prevention Applications Network
VA: Department of Veterans Affairs
VHA: Veterans Health Administration
VISN: Veteran Integrated Service Network
VSSC: VHA Support Service Center

## References

[1] McCarthy JF, Valenstein M, Kim HM, Ilgen MA, Zivin K, Blow FC (2009). Suicide mortality among patients receiving care in the Veterans Health Administration health system. Am J Epidemiol.

[2] McCarthy JF, Blow FC, Ignacio RV, Ilgen MA, Austin KL, Valenstein M (2012). Suicide among patients in the Veterans Affairs health system: rural–urban differences in rates, risks, and methods. Am J Public Health.

[3] Stone DM, Simon TR, Fowler KA, Kegler SR, Yuan K, Holland KM (2018). Vital signs: trends in state suicide rates — United States, 1999–2016 and circumstances contributing to suicide — 27 states, 2015. MMWR Morb Mortal Wkly Rep.

[4] Ursano RJ, Kessler RC, Naifeh JA, Herberman Mash HB, Nock MK, Aliaga PA (2018). Risk factors associated with attempted suicide among US Army soldiers without a history of mental health diagnosis. JAMA Psychiatry.

[5] Reger MA, Smolenski DJ, Carter SP (2018). Suicide prevention in the US Army: a mission for more than mental health clinicians. JAMA Psychiatry..

[6] Mott JM, Grubbs KM, Sansgiry S, Fortney JC, Cully JA (2015). Psychotherapy utilization among rural and urban veterans from 2007 to 2010. J Rural Health.

[7] Kirchner JE, Farmer MS, Shue VM, Blevins D, Sullivan G (2011). Partnering with communities to address the mental health needs of rural veterans. J Rural Health.

[8] Hogan MF, Goldstein GJ (2016). Suicide prevention: an emerging priority for health care. Health Aff.

[9] Joint Commission (2010). A follow-up report on preventing suicide: focus on medical/surgical units and the emergency department. JC Sentinel Event Alert Issue.

[10] National Action Alliance for Suicide Prevention. National Action Alliance for Suicide Prevention Research Prioritization Task Force [Internet]. 2017. Available from: http://actionallianceforsuicideprevention.org/research-prioritization-task-force

[11] Suicide Prevention Resource Center (SPRC). Zero suicide: transitions [Internet]. 2017. Available from: http://zerosuicide.sprc.org/toolkit/transition

[12] Wang Y-C, Hsieh L-Y, Wang M-Y, Chou C-H, Huang M-W, Ko H-C. Coping card usage can further reduce suicide reattempt in suicide attempter case management within 3-month intervention. Suicide and Life-Threatening Behavior. 2015 Jul;n/a-n/a. 10.1111/sltb.12177 26201436

[13] Offson M, Marcus SC, Bridge JA (2014). Focusing suicide prevention on periods of high risk. JAMA..

[14] Arias SA, Miller I, Camargo CA, Sullivan AF, Goldstein AB, Allen MH (2016). Factors associated with suicide outcomes 12 months after screening positive for suicide risk in the emergency department. Psychiatr Serv.

[15] USDHHS Office of the Surgeon General and National Action Alliance for Suicide Prevention. 2012 National strategy for suicide prevention: goals and objectives for action. Washington, DC: DHHS; 2012. 23136686

[16] Owens PL, Mutter R, Stocks C (2010). Healthcare cost and utilization project statistical brief 92: mental health and substance abuse-related emergency department visits among adults, 2007.

[17] Ting SA, Sullivan AF, Boudreaux ED, Miller I, Camargo CA (2012). Trends in U.S. emergency department visits for attempted suicide and self inflicted injury, 1993-2008. Gen Hosp Psychiatry.

[18] Motto JA (1976). Suicide prevention for high-risk persons who refuse treatment. Suicide Life Threat Behav.

[19] Motto JA, Bostrom AG (2001). A randomized controlled trial of postcrisis suicide prevention. Psychiatr Serv.

[20] Carter GL (2005). Postcards from the EDge project: randomised controlled trial of an intervention using postcards to reduce repetition of hospital treated deliberate self poisoning. BMJ..

[21] Carter GL, Clover K, Whyte IM, Dawson AH, D’este C (2007). Postcards from the EDge: 24-month outcomes of a randomised controlled trial for hospital-treated self-poisoning. Br J Psychiatry.

[22] Hassanian-Moghaddam H, Sarjami S, Kolahi A-A, Carter GL (2011). Postcards in Persia: randomised controlled trial to reduce suicidal behaviours 12 months after hospital-treated self-poisoning. Br J Psychiatry.

[23] Darke S, Ross J, Lynskey M, Teesson M (2004). Attempted suicide among entrants to three treatment modalities for heroin dependence in the Australian treatment outcome study (ATOS): prevalence and risk factors. Drug Alcohol Depend.

[24] Tarrier N, Barrowclough C, Andrews B, Gregg L (2004). Risk of non-fatal suicide ideation and behaviour in recent onset schizophrenia. Soc Psychiatr Psychiatr Epidemiol.

[25] Duberstein PR, Conwell Y, Conner KR, Eberly S, Evinger JS, Caine ED (2004). Poor social integration and suicide: fact or artifact? A case-control study. Psychol Med.

[26] O’Donnell L, O’Donnell C, Wardlaw DM, Stueve A (2004). Risk and resiliency factors influencing suicidality among urban African American and Latino youth. Am J Community Psychol.

[27] Nisbet PA (1996). Protective factors for suicidal black females. Suicide Life Threat Behav.

[28] Compton MT, Thompson NJ, Kaslow NJ (2005). Social environment factors associated with suicide attempt among low-income African Americans: the protective role of family relationships and social support. Soc Psychiatr Psychiatr Epidemiol.

[29] Thompson MP, Kaslow NJ, Short LM, Wyckoff S (2002). The mediating roles of perceived social support and resources in the self-efficacy-suicide attempts relation among African American abused women. J Consult Clin Psychol.

[30] Borowsky IW, Ireland M, Resnick MD (2001). Adolescent suicide attempts: risks and protectors. Pediatrics..

[31] Bearman PS, Moody J (2004). Suicide and friendships among American adolescents. Am J Public Health.

[32] Turvey CL, Conwell Y, Jones MP, Phillips C, Simonsick E, Pearson JL (2002). Risk factors for late-life suicide: a prospective, community-based study. Am J Geriatr Psychiatry.

[33] Wolk-Wasserman D (1986). Suicidal communication of persons attempting suicide and responses of significant others. Acta Psychiatr Scand.

[34] Neeleman JWS (1999). Ethnic minority suicide: a small area geographical study in South London. Psychol Med.

[35] Braucht GN (1979). Interactional analysis of suicidal behavior. J Consult Clin Psychol.

[36] Desai RA, Dausey DJ, Rosenheck RA (2005). Mental health service delivery and suicide risk: the role of individual patient and facility factors. Am J Psychiatry.

[37] Boggild AK, Heisel MJ, Links PS (2004). Social, demographic, and clinical factors related to disruptive behaviour in hospital. Can J Psychiatr.

[38] Reger MA, Luxton DD, Tucker RP, Comtois KA, Keen AD, Landes SJ, et al. Implementation methods for the Caring Contacts suicide prevention intervention. Professional Psychology: Research and Practice [Internet]. 2017 [cited 2017 Sep 4]; Available from: http://doi.apa.org/getdoi.cfm?doi=10.1037/pro0000134

[39] Luxton DD, Kinn JT, June JD, Pierre LW, Reger MA, Gahm GA (2012). Caring letters project: a military suicide-prevention pilot program. Crisis..

[40] Comtois Katherine Anne, Kerbrat Amanda H., DeCou Christopher R., Atkins David C., Majeres Justine J., Baker Justin C., Ries Richard K. (2019). Effect of Augmenting Standard Care for Military Personnel With Brief Caring Text Messages for Suicide Prevention. JAMA Psychiatry.

[41] Reger MA, Gebhardt HM, Lee JM, Ammerman BA, Tucker RP, Matarazzo BB, et al. Veteran preferences for the caring contacts suicide prevention intervention. Suicide Life-Threatening Behavior. 2018; https://onlinelibrary.wiley.com/doi/pdf/10.1111/sltb.12528. 10.1111/sltb.12528 30451311

[42] Luxton DD, June JD, Comtois KA (2013). Can postdischarge follow-up contacts prevent suicide and suicidal behavior?: a review of the evidence. Crisis.

[43] Denchev Peter, Pearson Jane L., Allen Michael H., Claassen Cynthia A., Currier Glenn W., Zatzick Douglas F., Schoenbaum Michael (2018). Modeling the Cost-Effectiveness of Interventions to Reduce Suicide Risk Among Hospital Emergency Department Patients. Psychiatric Services.

[44] Beautrais AL, Gibb SJ, Faulkner A, Fergusson DM, Mulder RT (2010). Postcard intervention for repeat self-harm: randomised controlled trial. Br J Psychiatry.

[45] Chen H, Mishara BL, Liu XX (2010). A pilot study of mobile telephone message interventions with suicide attempters in China. Crisis..

[46] Berrouiguet S, Gravey M, Le Galudec M, Alavi Z, Walter M (2014). Post-acute crisis text messaging outreach for suicide prevention: a pilot study. Psychiatry Res.

[47] Department of Veterans Affairs. QUERI – Quality Enhancement Research Initiative [Internet]. [cited 2018 May 30]. Available from: https://www.queri.research.va.gov/

[48] Harvey G, Kitson AL (2015). Implementing evidence-based practice in healthcare: a facilitation guide.

[49] Kilbourne AM, Abraham KM, Goodrich DE, Bowersox NW, Almirall D, Lai Z (2013). Cluster randomized adaptive implementation trial comparing a standard versus enhanced implementation intervention to improve uptake of an effective re-engagement program for patients with serious mental illness. Implement Sci.

[50] Kirchner JE, Ritchie MJ, Pitcock JA, Parker LE, Curran GM, Fortney JC (2014). Outcomes of a partnered facilitation strategy to implement primary care–mental health. J Gen Intern Med.

[51] Stetler CB, Legro MW, Rycroft-Malone J, Bowman C, Curran G, Guihan M (2006). Role of “external facilitation” in implementation of research findings: a qualitative evaluation of facilitation experiences in the Veterans Health Administration. Implement Sci.

[52] Ritchie MJ, Dollar KM, Kearney LK, Kirchner JE (2014). Responding to needs of clinical operations partners: transferring implementation facilitation knowledge and skills. Psychiatr Serv.

[53] Harvey G, Loftus-Hills A, Rycroft-Malone J, Titchen A, Kitson A, McCormack B (2002). Getting evidence into practice: the role and function of facilitation. J Adv Nurs.

[54] Fortney JC, Pyne JM, Smith JL, Curran GM, Otero JM, Enderle MA (2009). Steps for implementing collaborative care programs for depression. Popul Health Manag.

[55] Department of Veterans Health Administration, Health Services Research & Development, Quality Enhancement Research Initiative. Implementation Guide [Internet]. 2013 [cited 2019 Jun 4]. Available from: https://www.queri.research.va.gov/implementation/ImplementationGuide.pdf

[56] Ritchie MJ, Dollar KM, Miller CJ, Oliver KA, Smith JL, Lindsay JA, et al. Using implementation facilitation to improve care in the Veterans Health Administration (Version 2) [Internet]. Veterans Health Administration, Quality Enhancement Research Initiative (QUERI) for team-based behavioral health; 2017. Available from: https://www.queri.research.va.gov/tools/implementation/Facilitation-Manual.pdf

[57] Glasgow RE, Vogt TM, Boles SM (1999). Evaluating the public health impact of health promotion interventions: the RE-AIM framework. Am J Public Health.

[58] Department of Veterans Affairs. Strategic Analytics for Improvement and Learning (SAIL) [Internet]. [cited 2018 May 30]. Available from: https://www.va.gov/qualityofcare/measure-up/strategic_analytics_for_improvement_and_learning_sail.asp

[59] Hamilton AB. Qualitative methods in rapid turn-around health services research. 2013 Dec 11; HSR&D Center for the Study of Healthcare Innovation, Implementation & Policy VA Greater Los Angeles Healthcare System.

[60] Bernard HR, Wutich A, Ryan GW (2016). Analyzing qualitative data: systematic approaches.

